# *Firmicutes* and *Blautia* in gut microbiota lessened in chronic liver diseases and hepatocellular carcinoma patients: a pilot study

**DOI:** 10.1080/21655979.2021.1982273

**Published:** 2021-10-19

**Authors:** Tianyou Chen, Rongrong Ding, Xiaorong Chen, Yunfei Lu, Jia Shi, Ying Lü, Bozong Tang, Wensi Zhang, Chen Ye, Min Yuan, Zongguo Yang

**Affiliations:** a Department of Interventional Medicine, Shanghai Public Health Clinical Center, Fudan University, Shanghai, China; b Department of Hepatobiliary Medicine, Shanghai Public Health Clinical Center, Fudan University, Shanghai, China; c Department of Integrative Medicine, Shanghai Public Health Clinical Center, Fudan University, Shanghai, China

**Keywords:** Gut microbiota, liver disease, hepatocellular carcinoma, Firmicutes, Blautia

## Abstract

The gut microbiota system plays a vital role in liver diseases. This study aimed to address the diversity of gut microbiota and its correlations with clinical parameters in healthy individuals, chronic liver disease (CLD), and hepatocellular carcinoma (HCC) patients. Fecal specimens of nine healthy individuals, 11 CLD, and 21 HCC were collected. The diversity of gut microbiota was examined by PCR and Illumina MiSeq sequencing and analyzed using 16S rRNA gene sequencing database. The correlations between gut microbiota and the clinical parameters of participants were also addressed. Compared to healthy individuals, *Firmicutes* at a phylum level decreased in CLD and HCC patients and *Proteobacteria* increased (p < 0.05). The composition of *Blautia* on a genus level in CLD and HCC patients significantly decreased compared to healthy controls (p < 0.05). *Firmicutes* composition was negatively associated with age and number of males (p < 0.05) and was positively associated with monocytes, high-density lipoprotein cholesterol (HDL-C), and estimated glomerular filtration rate (eGFR) levels (p < 0.05). At a genus level, *Blautia* composition was negatively associated with cirrhosis, age, and number of males (p < 0.01), while it was positively associated with red blood cells (RBCs), triglycerides, HDL-C, and lymphocyte levels (p < 0.05). Conclusively, there was a significant compositional difference in gut microbiota in CLD and HCC patients compared with healthy subjects. *Firmicutes* and *Blautia* in gut microbiota system lessened in CLD and HCC patients. Clinical biochemical parameters have an impact on the diversity of gut microbiota in liver diseases.

## 1. Introduction

The gut microbiome is present in the intestinal system and produces metabolites that exert various supporting effects on host biological functions [1]. Dysbiotic microbiota can affect the host’s immune system and mucosal integrity through multiple mechanisms involving modulation of inflammasome signalings and regulation of immune cell functions and responses [2]. Currently, it is unclear whether the dysbiotic microbiota is a cause of diseases or a consequence of disease-related changes. However, microbiota transplantation is a promising therapeutic approach for some pathological phenotypes, suggesting a causative relationship between gut microbiota and disease pathogenesis [3–6].

In view of the close links between the gut system and the liver, many pathological processes have been addressed in light of a microbe-centered hypothesis of liver damage [7]. Changes in the composition and function of gut microbiota are correlated with the development and progression of multiple kinds of liver diseases [8,9]. Many studies have focused on the links of noninfectious variables and dysbiotic microbiota in liver diseases. In a report by Lu et al., the authors found that the densities of *Bifidobacteria* and *Lactobacillus* were significantly decreased and those of the *Enterococcus* and *Enterobacteriaceae* were significantly increased in chronic hepatitis B (CHB) patients compared to healthy controls [10]. In addition, the intestinal bacterial products and gut microbiota imbalance can promote progression to cirrhosis and hepatocellular carcinoma (HCC) [11–13]. Unfortunately, most literature identified the pathological changes of gut microbiota in the progression of nonviral HCC patients [1,9].

Since chronic liver diseases, particularly chronic hepatitis B virus (HBV) infection, have been demonstrated to have an impact on cirrhosis and tumorigenesis, this study aims to investigate the diversity of gut microbiota in healthy individuals, chronic liver diseases (mainly CHB subjects), and HCC patients. This study wants to compare the gut microbiota composition in different liver diseases, in the hope of gaining a deep understanding of the disease progression and cancer development.

## 2. Materials and methods

### 2.1. Ethic statement

All participants provided the written informed consent. The protocol and informed consent documents were reviewed and approved by the Ethics Committee, Shanghai Public Health Clinical Center, Fudan University (No. 2020-S117-01).

### 2.2. Participants

Nine healthy individuals, 11 chronic liver diseases (CLD), and 21 HCC in Shanghai Public Health Clinical Center, Fudan University, were included in this pilot study. The healthy individuals met the following criteria: 1) normal blood /urine/stool routine tests and liver-kidney functions, 2) no history of liver disease, and 3) no intestinal probiotics and antibiotics prescriptions within 2 weeks before sample collection. CLD patients included chronic hepatitis and cirrhosis patients, regardless of the disease status and liver functions. HCC was either diagnosed pathologically or radiologically according to guidelines for diagnosis and treatment of primary liver cancer in China – two of the four imaging studies, including computed tomography (CT), magnetic resonance imaging (MRI), gadolinium-ethoxybenzyl-diethylenetriamine pentaacetic acid MRI, or contrast-enhanced ultrasonography, showing an arterial enhanced mass less than 2 cm, or one of the four imaging studies above showing an arterial enhanced mass greater than 2 cm [14]. Subjects with acute or chronic gastrointestinal diseases, renal failure, liver failure, sepsis, autoimmune disorders, and other uncontrolled life-threatening diseases were excluded from the study. Fecal samples of all the participants were collected with germ-free collection boxes. The fecal samples were stored in −80°C refrigerator immediately.

### 2.3. Laboratory examinations

Blood routine tests and liver-kidney functions of participants were conducted by the Medical Laboratory on the basis of the Standard Operation Procedure for medical examinations in Shanghai Public Health Clinical Center, Fudan University.

### 2.4. DNA extraction and PCR amplification

The genomic DNA of the microbial community was extracted from all these samples using a TransStart® FastPfu DNA Polymerase system (TransGen AP221-02, TransGen Biotech, Beijing, China) following the manufacturer’s instructions. The DNA extract was detected on 1% agarose gel, and DNA concentration and purity were determined using a NanoDrop 2000 UV-vis spectrophotometer (Thermo Scientific, Wilmington, USA). The hypervariable regions V3-V4 of the bacterial 16S rRNA gene were amplified using an ABI GeneAmp® 9700 PCR thermocycler (ABI, CA, USA) with primer pairs 338 F (5ʹ- ACTCCTACGGGAGGCAGCAG −3ʹ) and 806 R (5ʹ- GGACTACHVGGGTWTCTAAT −3ʹ). The PCR amplification of the 16S rRNA gene was conducted according to the procedures [15,16]. PCR reactions were performed in triplicate. According to the manufacturer’s instructions, the PCR product was extracted from 2% agarose gel and purified by the AxyPrep DNA Gel Extraction Kit (Axygen Biosciences, Union City, CA, USA) and quantified using Quantus™ Fluorometer (Promega, USA).

### 2.5. Illumina MiSeq sequencing

The purified amplified products were combined on the Illumina MiSeq PE300 platform/NovaSeq PE250 platform (Illumina, San Diego, USA) by isomolar and paired-terminal sequencing according to the standard protocols by Shanghai Majorbio Bio-Pharm Technology Co. Ltd. (Shanghai, China).

### 2.6. Processing of sequencing data

The original 16S rRNA gene sequencing readings were demultiplexed, quality-filtered using fastp version 0.20.0 [17], and merged using FLASH version 1.2.7 [18] according to the criteria reported by Wang et al. [19].

Operational taxonomic units (OTUs) with 97% similarity cutoff [20,21] were clustered using UPARSE version 7.1 [20], and chimeric sequences were identified and deleted. The classification of each OTU representative sequence was computed using RDP Classifier version 2.2 [22] with a confidence threshold of 0.7 for the 16S rRNA database.

### 2.7. Statistical analysis

The normally distributed continuous data were described by mean ± standard deviation (SD) and the enumeration data was described by the frequency with percentage. Student’s t-test and chi-square test based on variable types are used to analyze the differences of variables between groups. The relative abundance was used to compute the density of gut microbiota. Principal coordinate analysis (PCoA) was used to compare the composition of gut microbiota between groups. The distance in gut microbiota composition between samples was calculated using the Bray-Curtis dissimilarity matrix. Spearman correlation analysis between clinical data and gut microbiota was also performed. Stata v16.1 (Stata, Texas, USA) was used. A two-side p < 0.05 were considered significance.

## 3. Results

Using 16S rRNA sequencing analysis, a significant compositional difference in gut microbiota was observed in CLD and HCC patients compared with healthy subjects. Compared to healthy individuals, *Firmicutes* at a phylum level decreased in CLD and HCC patients and *Proteobacteria* increased. The composition of *Blautia* on a genus level in CLD and HCC patients significantly decreased compared to healthy controls. *Firmicutes* and *Blautia* compositions were affected by multiple clinicopathological factors, for instance, age, gender, and HDL-C. The current study preliminarily indicated that alterations of gut microbiota might be involved in the progression of liver diseases.

### 3.1. Participant characteristics

As summarized in Table 1, one male was in the healthy control group, seven in the CLD group, and 21 in the HCC group (p < 0.05). The average age of healthy individuals, CLD and HCC patients were 29.2 years, 44.5 years, and 62.4 years, respectively (p < 0.05). Most HCC patients suffered from cirrhosis compared to healthy controls and CLD patients (p < 0.05). The levels of red blood cells (RBC), hemoglobin, platelets, and lymphocytes in blood routine tests were significantly low in HCC patients (p < 0.05). By concerning liver functions, the levels of alanine aminotransferase (ALT), aspartate aminotransferase (AST), alkaline phosphatase (ALP), and gamma-glutamyl transferase (GGT) were significantly higher in CLD and HCC patients compared to healthy individuals (p < 0.05). On the contrary, the albumin levels of CLD and HCC patients were significantly decreased compared to healthy controls (p < 0.05). The serum levels of triglycerides and total cholesterol were also significantly different in these three groups (p < 0.05), and the levels of HDL-C and LDL-C gradually decreased in the healthy controls, CLD and HCC patients (p < 0.05). In addition, the fasting plasma glucose (FPG) levels gradually increased from healthy individuals, CLD subjects to HCC patients (p < 0.05). No significances of the urea, creatinine and eGFR were observed in these three groups (p > 0.05).

**Table 1.** Baseline characteristics of participants included in this study

Variables	Normal (n = 9)	CLD (n = 11)	HCC (n = 21)	p value
Male, n (%)	1 (11.1)	7 (63.6)	21 (100)	<0.05
Age, years, mean ± SD	29.2 ± 8.5	44.5 ± 7.7	62.4 ± 10.4	<0.05
HBV infection, n (%)	0	9 (81.8)	12(57.1)	>0.05^‡^
Cirrhosis, n (%)	0	1 (9.1)	18 (85.7)	<0.05^‡^
Ascites, n (%)	0	1 (9.1)	0	>0.05^‡^
Intestinal microecological agents, n (%)	0	2 (18.2) ^†^	0	>0.05^‡^
Disease history, n (%)				
Diabetes	0	1 (9.1)	2 (9.5)	>0.05^‡^
Fatty liver diseases	0	1 (9.1)	0	>0.05^‡^
Non-HCC Cancer	0	0	2 (9.5)	>0.05^‡^
WBC, 10^3^/mm^3^, mean ± SD	5.8 ± 1.7	5.1 ± 1.4	5.3 ± 2.1	>0.05
RBC, 10^4^/mm^3^, mean (SD)	4.5 ± 0.3	4.6 ± 0.6	4.0 ± 0.8	<0.05
Hemoglobin, g/L, mean (SD)	133. 8 ± 10.7	141.7 ± 17.3	124.6 ± 19.1	<0.05
Platelet, 10^3^/mm^3^, mean (SD)	260.7 ± 49.7	198.2 ± 88.0	112.3 ± 79.7	<0.05
Neutrophils, 10^3^/mm^3^, median (IQR)	2.6 (2.4, 3.7)	2.8 (2.0, 3.3)	2.8 (2.1, 4.5)	>0.05
Lymphocytes, 10^3^/mm^3^, mean (SD)	1.97 ± 0.39	1.81 ± 0.8	1.23 ± 0.69	<0.05
Monocytes, 10^3^/mm^3^, mean (SD)	0.42 ± 0.1	0.42 ± 0.13	0.52 ± 0.17	>0.05
ALT, U/L, median (IQR)	11 (9, 17)	30 (18, 315)	59.5 (22, 116)	<0.05
AST, U/L, median (IQR)	16 (14, 19)	44 (16, 126)	35 (24, 60)	<0.05
AKP, U/L, median (IQR)	58 (55, 69)	79 (60, 115)	101.5 (78, 172)	<0.05
GGT, U/L, median (IQR)	15 (11, 17)	40 (18, 97)	31 (18, 193)	<0.05
LDH, U/L, median (IQR)	177 (155, 184)	198 (174, 257)	207 (177, 323)	>0.05
TBiL, μmol/L, median (IQR)	11.5 (9.6, 19)	15.6 (11.8, 31)	16.6 (10.9, 29.9)	>0.05
DBiL, μmol/L, median (IQR)	4.4 (3.8, 6.5)	7 (4.7, 12.6)	6.8 (4.5, 10.9)	>0.05
Albumin, g/L, mean (SD)	46.8 ± 2.0	40.5 ± 5.9	36.2 ± 4.1	<0.05
Globulin, g/L, mean (SD)	30.4 ± 2.7	30.1 ± 5.6	26.5 ± 5.2	>0.05
Triglycerides, mmol/L, median (IQR)	0.8 (0.6, 0.9)	1.4 (1.0, 2.8)	0.8 (0.7, 0.8)	<0.05
Total cholesterol, mmol/L, mean (SD)	5.1 ± 0.6	4. 6 ± 1.0	3.3 ± 0.6	<0.05
HDL-C, mmol/L, mean (SD)	1.6 ± 0.2	1.2 ± 0.4	1.1 ± 0.2	<0.05
LDL-C, mmol/L, mean (SD)	3.4 ± 0.6	3.0 ± 1.0	1.7 ± 0.4	<0.05
Urea, mmol/L, mean (SD)	4.47 ± 1.36	4.74 ± 1.35	4.93 ± 1.37	>0.05
Creatinine, μmol/L, median (IQR)	54.2 (48.2, 58.6)	65.9 (55.5, 72.6)	60.5 (51.0, 76.8)	>0.05
FPG, mmol/L, median (IQR)	4.8 (4.4, 5.6)	5.4 (5.0, 5.6)	7.0 (5.0, 11.3)	<0.05
eGFR, mean (SD)	125.8 ± 14.8	117.5 ± 16.4	118.9 ± 37.3	>0.05

HBV, hepatitis B virus; WBC, white blood cells; RBC, red blood cells; ALT, alanine aminotransferase; AST, aspartate aminotransferase; AKP, alkaline phosphatase; GGT, gamma-glutamyl transferase; LDH, lactate dehydrogenase; TBiL, total bilirubin; DBiL direct bilirubin; HDL-C, high density lipoprotein cholesterol; LDL-C, low density liptein cholesterol; FPG, Fasting plasma glucose; eGFR, estimated glomerular filtration rate.

^†^One received clostridium butyricum capsules and one received clostridium butyricum capsules combined with lactobacillus acidophilus tablets.

^‡^p value of comparison between CLD and HCC.

### 3.2. Diversity of gut microbiota on phylum and genus levels

**Figure 1.**
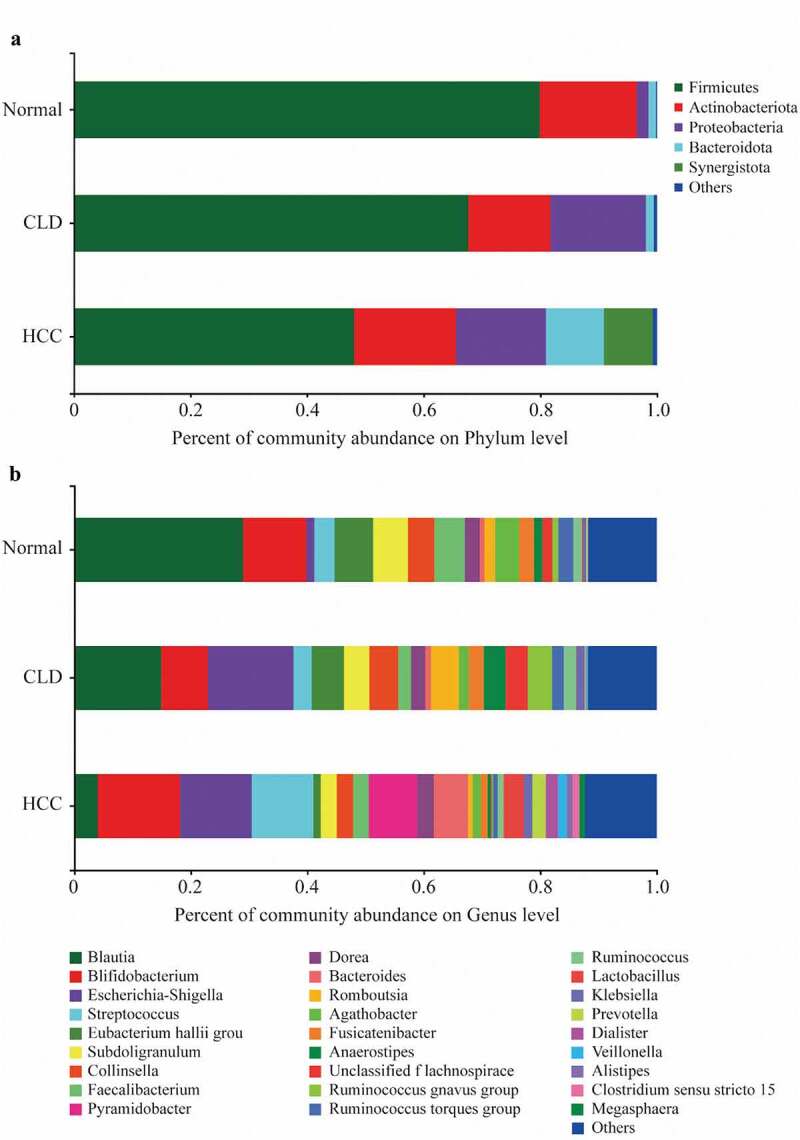
Percentage of gut microbiota on phylum level (a) and genus level (b)

As shown in Figure 1, *Firmicutes* were more abundant in healthy individuals and decreased in CLD and HCC patients at phylum level, while the percentage of *Proteobacteria* increased in CLD and HCC patients compared to healthy controls (Figure 1a). In addition, *Bacteroidota* and *Synergistota* were more abundant in HCC patients compared to healthy controls and CLD patients (Figure 1a). At genus level, *Blautia* was more abundant in healthy individuals, while it decreased in CLD patients and much lower in HCC patients (Figure 1b). The Circos diagrams of gut microbiota composition in healthy individuals, CLD, and HCC patients at phylum and genus levels are given in Figure S1 and Figure S2, respectively.

### 3.3. Comparison of gut microbiota composition

**Figure 2.**
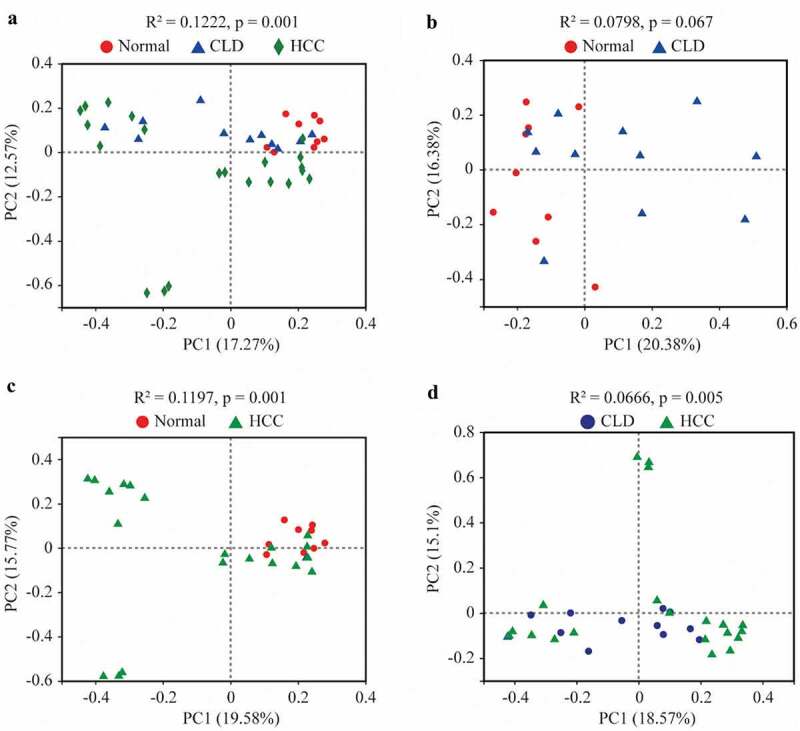
Principal co-ordinates analysis (PCoA) of gut microbiota among healthy individuals, CLD and HCC patients

As shown in Figure 2, the compositions of gut microbiota in healthy individuals, CLD, and HCC patients were significantly different (Adonis, p = 0.001, Figure 2a). The composition of the microbiome in HCC patients was significantly different from that in healthy individuals and CLD patients (Adonis, p = 0.001 and p = 0.005, respectively, Figure 2c and d). No significance of gut microbiota composition was found between healthy individuals and CLD patients (Adonis, p = 0.067, Figure 2b).

**Figure 3.**
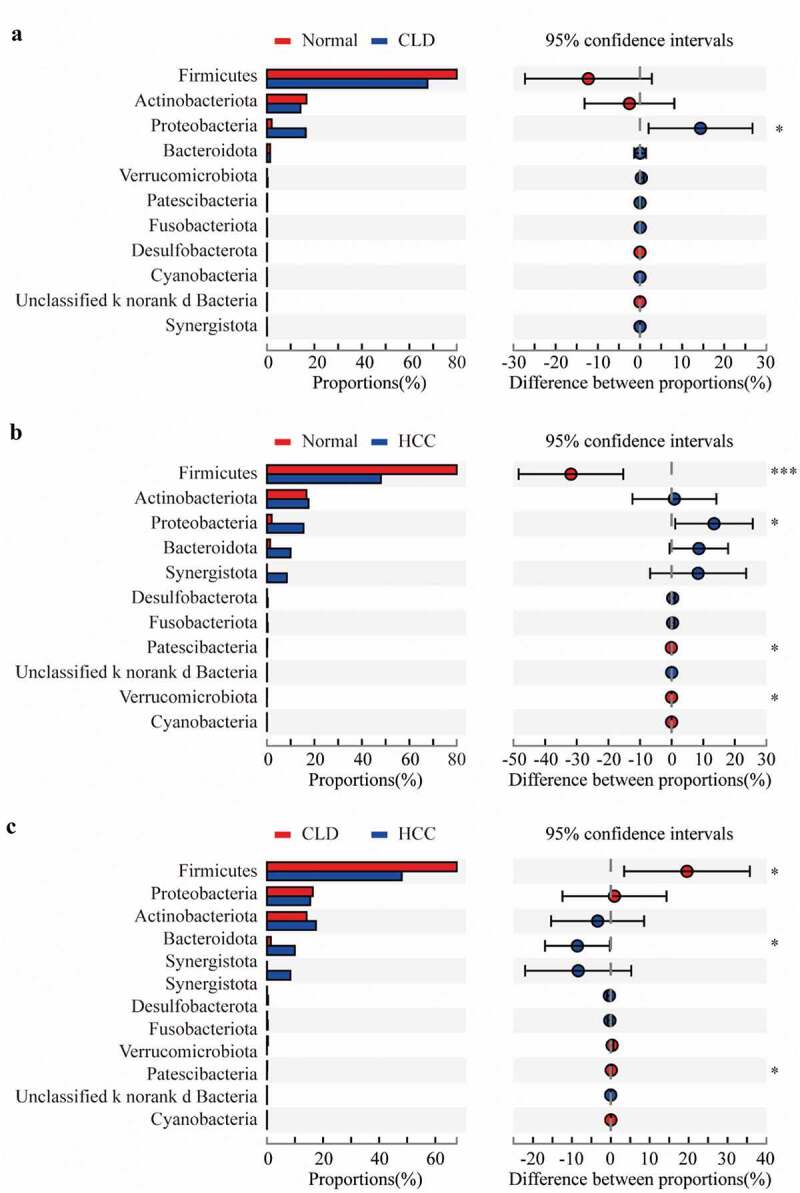
Proportion comparisons of gut microbiota between healthy individuals and CLD patients (a), healthy individuals and HCC patients (b), and CLD and HCC patients (c) on phylum level

At the phylum level, *Proteobacteria* had a significantly higher proportion in both CLD and HCC patients compared to healthy individuals (both p < 0.05, Figure 3a and b). Additionally, the community composition of *Firmicutes* was significantly decreased in HCC patients compared to healthy individuals (p < 0.001, Figure 3b) and CLD patients (p < 0.05, Figure 3c). With a relatively lower proportion, *Patescibacteria* was differentially distributed between HCC patients and healthy controls/CLD patients (both p < 0.05, Figures 3b and c); *Verrucomicrobiota* was also significantly different between HCC patients and healthy controls (p < 0.05, Figure 3b).

**Figure 4.**
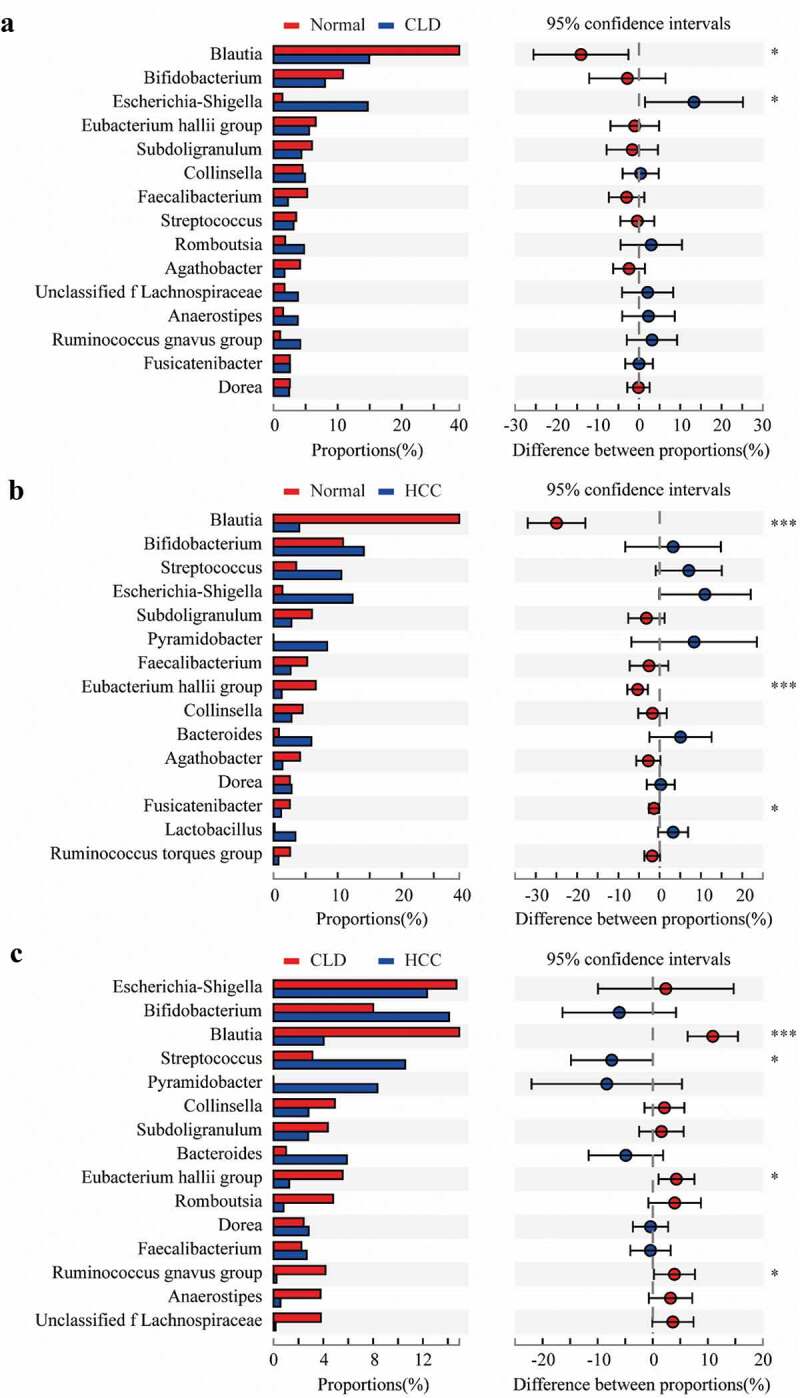
Proportion comparisons of gut microbiota between healthy individuals and CLD patients (a), healthy individuals and HCC patients (b), and CLD and HCC patients (c) on genus level

Compared to healthy individuals, the composition of *Blautia* at genus level in CLD and HCC patients significantly decreased (p < 0.05 and p < 0.001, respectively, Figures 4a and b); *Escherichia-Shigella* proportion significantly increased in CLD patients (p < 0.05, Figure 4a); *Eubacterium hallii group* and *Fusicatenibacter* were significantly lower in HCC patients (p < 0.001 and p < 0.05, respectively, Figure 4b). Compared to CLD patients, proportions of *Blautia, Eubacterium hallii group*, and *Ruminococcus gnavus* group were significantly lower in HCC patients (all p < 0.05, Figure 4c). Conversely, the proportion of *Streptococcus* was significantly higher in HCC patients compared to CLD cases (p < 0.05, Figure 4c).

### 3.4. Correlations between gut microbiota and clinical biochemical parameters

Multicollinearity of the clinical biochemical parameters was checked by calculating the Variance Inflation Factor (VIF). All variables with a VIF value of less than eight were included in the correlation analysis. After VIF analysis, variables, namely, male, age, HBV infection, cirrhosis, ascites, intestinal microecological agents, RBC, neutrophils, lymphocytes, monocytes, AST, GGT, direct bilirubin (DBiL), triglycerides, HDL-C, urea, creatinine, FPG, and eGFR, were taken for the Spearman correlation model (Supplementary Table S1).

**Figure 5.**
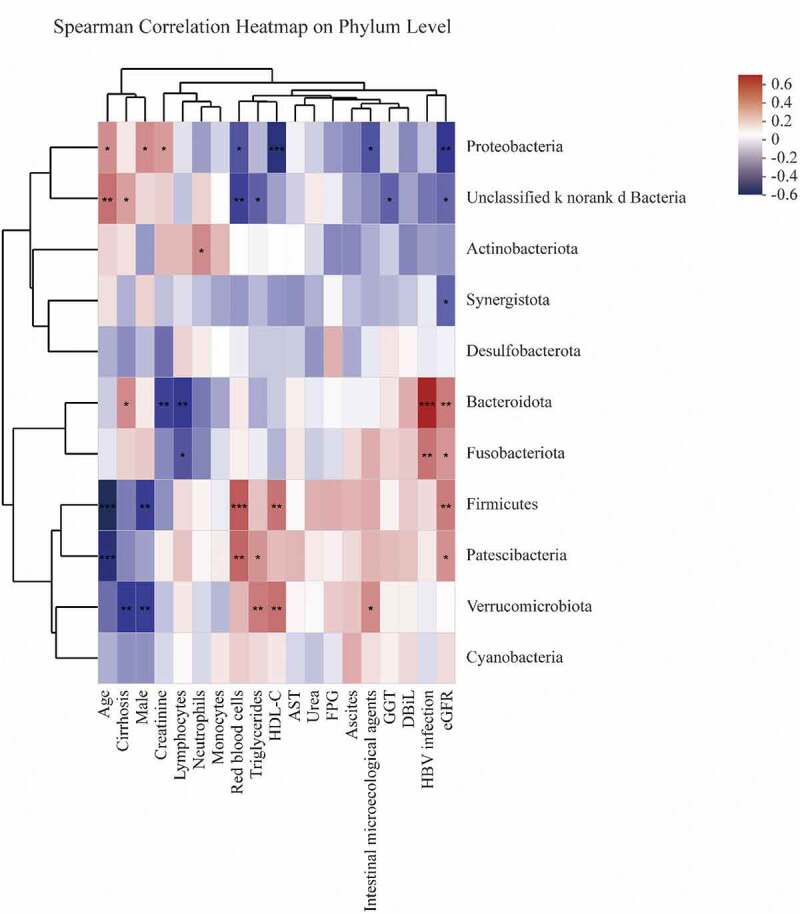
Spearman correlations between gut microbiota and clinical biochemical parameters on phylum level

As detailed in Figure 5, *Firmicutes* composition was negatively associated with age and male numbers (p < 0.01, Figure 5) and was positively associated with monocytes, HDL-C, and eGFR levels (p < 0.05, Figure 5). *Proteobacteria* were negatively correlated with RBC, HDL-C, intestinal microecological agents, and eGFR levels (p < 0.05, Figure 5) and were positively correlated with age, male numbers, and creatine (p < 0.05, Figure 5). *Bacteroidota* was negatively associated with creatine and lymphocytes and was positively associated with cirrhosis, HBV infection, and eGFR (p < 0.05, Figure 5). *Fusobacteriota* was negatively correlated with lymphocytes and positively correlated with HBV infection and eGFR (p < 0.05, Figure 5). *Patescibacteria* was negatively associated with age and positively associated with RBC, triglycerides, and eGFR (p < 0.05, Figure 5). *Verrucomicrobiota* was conversely correlated with cirrhosis and male numbers and positively correlated with triglycerides, HDL-C, and intestinal microecological agents (p < 0.05, Figure 5).

**Figure 6.**
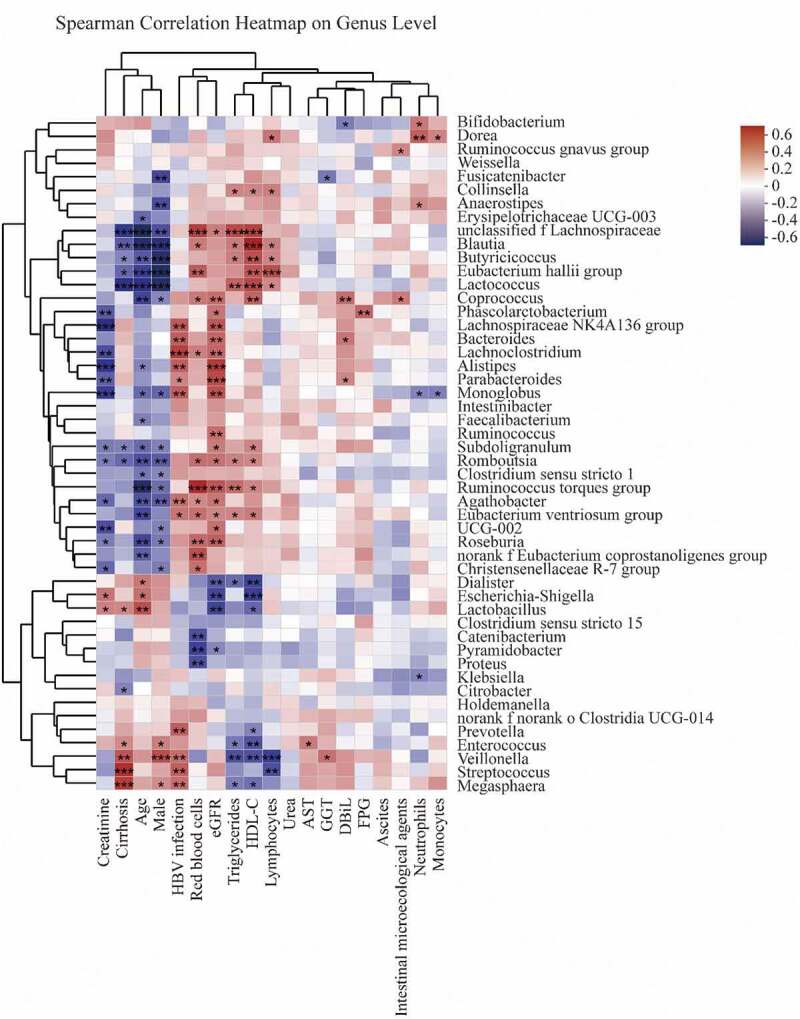
Spearman correlations between gut microbiota and clinical biochemical parameters on genus level

The Spearman correlations of the gut microbiome at genus level and variables are described in Figure 6. *Blautia* composition was negatively associated with cirrhosis, age, and male (p < 0.01, Figure 6), while it was positively associated with RBC, triglycerides, HDL-C, and lymphocyte levels (p < 0.05, Figure 6). Additionally, HBV infection positively contributed to increasing proportions of *Megasphaera, Streptococcus, Veillonella, Prevotella, Eubacterium ventriosum group, Agathobacter, Monoglobus, Parabacteroides, Alistipes, Lachnoclostridium, Bacteroides*, and *Lachnospiraceae NK4A136 group* (p < 0.05, Figure 6).

## 4. Discussion

A balanced gut microbiota provides essential nutrients, maintains intestinal mucosal integrity, protects the body from pathogens, produces antimicrobial peptides, and plays an active role in the innate and adaptive immune system [23,24]. The imbalance of the gut microbiota leads to an increased risk of endotoxemia, insulin resistance, systemic inflammation, obesity and metabolic disorders, nonalcoholic fatty liver diseases, and cancer [23–26]. Currently, probiotics and prebiotics can restore the normal flora of the gut microbiome and show favorable efficacy in the treatment of some pathological conditions including liver diseases [9,27,28]. The present study partly supplements the understanding of gut microbiota in the development of CLD and HCC.

*Firmicutes* and *Bacteroidetes* are the most common phylum in the gut microbiota [23], and *Firmicutes* are more abundant than *Bacteroidetes* [29]. Organisms in *Firmicutes* phyla are an important part of normal flora. Many *Firmicutes* are able to produce endospores, which are strongly dehydrated, and highly resistant to environmental stresses, and can survive extreme conditions. In addition, *Firmicutes* can generate more abundant energy than *Bacteroidetes* [30]. The *Firmicutes* abundance was reported to be correlated with body mass index in healthy individuals [31,32]. Pathologically, *Firmicutes* could induce obesity and hepatic steatosis and promoted the elevation of TNF-α mRNA levels, suggesting that *Firmicutes* might be involved in the pathogenesis of the nonalcoholic fatty liver disease (NAFLD) [33–35]. Many studies have shown a significant increase in *Firmicutes* in patients with gallstone disease [36]. Moreover, increased *Firmicutes* has been reported in multiple sclerosis patients [37,38]. In contrast to elevated proportions of *Firmicutes* in these pathological phenotypes, the present study indicated that CLD and HCC patients had a remarkably low proportion of *Firmicutes*, which might lead to more sensitivity to environmental stresses and lack of energy. The current profile of *Firmicutes* could be a reference for deep investigations aimed at illustrating the contribution of this phylum to the progression of liver diseases and HCC.

As for lower taxa of the *Firmicutes, Blautia* has been shown to be significantly and negatively related to visceral fat accumulation in healthy Japanese people of men and women [39,40]. These findings partly explained that *Blautia* was positively associated with triglycerides and HDL-C in this study. *Blautia* is widely distributed in mammalian feces and intestines and has indicated a series of potential probiotic properties [41]. It plays a favorable role in alleviating inflammatory diseases and metabolic disorder, with antibacterial activities against specific microorganisms [41–43]. Emerging literature demonstrated that *Blautia* is of great importance in maintaining host health and correlating with physiological dysfunctions including cancer and various inflammatory diseases [41,44]. For instance, *Blautia* decreased significantly in diabetes both in children and adults compared to healthy controls [45,46], and increased abundance of *Blautia* was correlated with the improvements in glucose and lipid homeostasis [47]. Increased amounts of *Blautia* were associated with a reduced lethality and favorable survival in acute graft-versus-host disease after allogeneic blood/marrow transplantation in blood malignancies [48]. In the present report, *Blautia* was the highest in healthy individuals, low in CLD patients, and the lowest in HCC patients and inversely correlated with cirrhosis. Kakiyama et al. revealed that cirrhosis patients had a lower abundance of *Blautia* compared to healthy controls, which is inconsistent with the results of this study [49]. That is, *Blautia* should be a potential agent in the comprehensive treatment for liver diseases.

In the current report, *Firmicutes* and *Blautia* compositions were correlated with clinical biochemical parameters, such as age, male, triglycerides, HDL-C, RBC, monocytes, and lymphocyte levels. Part of these factors were supported by previous publications [33,36,41,50]. Notably, age and gender were differentially distributed in the healthy individuals and CLD and HCC patients in the current study. As reported previously, the gut microbiota community varied as age changed and was influenced by gender [51,52]. Considering the different distributions of variables among healthy individuals and CLD and HCC patients, the correlations between gut microbiota composition and clinical biochemical parameters should be reevaluated in the same populations using variable-matching methods in the future.

Some limitations exist in this study. First, this study focused on the phylum and genus levels and did not conduct in-depth studies at the species or even strain levels. Second, the sample size is relatively small and no tumor-free cirrhosis patients are included. Third, variables within each group and among the groups were differently distributed, like age and gender, and biases of factors correlating with gut microbiome existed. Although, the results preliminarily revealed the changes of gut microbiota during the development of liver diseases, reduction of *Firmicutes* and *Blautia* might worsen the severity of liver pathological conditions.

## 5. Conclusion

There was a significant compositional difference in gut microbiota in CLD and HCC patients compared with healthy subjects. Notably, *Firmicutes* at a phylum level and *Blautia* at a genus level significantly decreased in CLD and HCC patients compared with healthy individuals. *Firmicutes* and *Blautia* compositions were affected by multiple clinicopathological factors, for instance, age, gender, and HDL-C. The current study preliminarily indicated that changes in gut microbiota composition were involved in the progression of liver diseases. Reversing the imbalanced gut microbiota system might be a potential therapy in this population.

## Supplementary Material

Supplemental Material

## Data Availability

All the datasets were available from the corresponding author (Z. Yang) with reasonable request.

## Highlights

- The gut microbiota changed as the liver disease developed.
- There was a significant compositional difference in gut microbiota in CLD and HCC patients compared with healthy subjects.
- *Firmicutes* and *Blautia* lessened in CLD and HCC patients.
- The composition of gut microbiota is influenced by multiple clinicopathological factors.
