# Supplementary material for: Firmicutes and Blautia in gut microbiota lessened in chronic liver diseases and hepatocellular carcinoma patients: a pilot study
Source: Bioengineered. 2021 Oct 19;12(1):8233–46. doi: 10.1080/21655979.2021.1982273 (PMC8806631; doi:10.1080/21655979.2021.1982273)

**Supplementary Materials**

***Firmicutes* and *Blautia* in Gut Microbiota Lessened in Chronic Liver Diseases and Hepatocellular Carcinoma Patients: A Pilot Study**

**Table S1** Variance inflation factor (VIF) selection of clinical variables.

| Variables | Original-VIF | Post-VIF |
| --- | --- | --- |
| Male | 11.65 | 6.0 |
| Age | 19.82 | 5.11 |
| HBV infection | 13.32 | 2.49 |
| Cirrhosis | 20.21 | 5.43 |
| Ascites | 40.25 | 2.95 |
| Intestinal microecological agents | 81.59 | 4.06 |
| WBC | 1409.25 | - |
| RBC | 137.5 | 6.97 |
| Hemoglobin | 151.11 | - |
| Platelet | 25.87 | - |
| Neutrophils | 607.85 | 4.29 |
| Lymphocytes | 249.51 | 3.68 |
| Monocytes | 18.15 | 7.47 |
| ALT | 219.5 | - |
| AST | 183.8 | 2.33 |
| AKP | 19.42 | - |
| GGT | 22.43 | 2.74 |
| LDH | 36.67 | - |
| TBiL | 46.02 | - |
| DBiL | 50.25 | 1.99 |
| Albumin | 79.84 | - |
| Globulin | 30.23 | - |
| Triglycerides | 22.21 | 2.35 |
| Total cholesterol | 663.02 | - |
| HDL-C | 124.91 | 5.99 |
| LDL-C | 231.63 | - |
| Urea | 10.32 | 4.73 |
| Creatinine | 22.61 | 7.22 |
| FPG | 14.53 | 2.52 |
| eGFR | 29.46 | 4.33 |

HBV, hepatitis B virus; WBC, white blood cells; RBC, red blood cells; ALT, alanine aminotransferase; AST, aspartate aminotransferase; AKP, alkaline phosphatase; GGT, gamma-glutamyl transferase; LDH, lactate dehydrogenase; TBiL, total bilirubin; DBiL direct bilirubin; HDL-C, high density lipoprotein cholesterol; LDL-C, low density liptein cholesterol; FPG, Fasting plasma glucose; eGFR, estimated glomerular filtration rate.

**Supplementary Figure legends**

**Figure S1** The Circos diagram of gut microbiota composition in healthy individuals, CLD and HCC patients on phylum level.

**Figure S2** The Circos diagram of gut microbiota composition in healthy individuals, CLD and HCC patients on genus levels

**
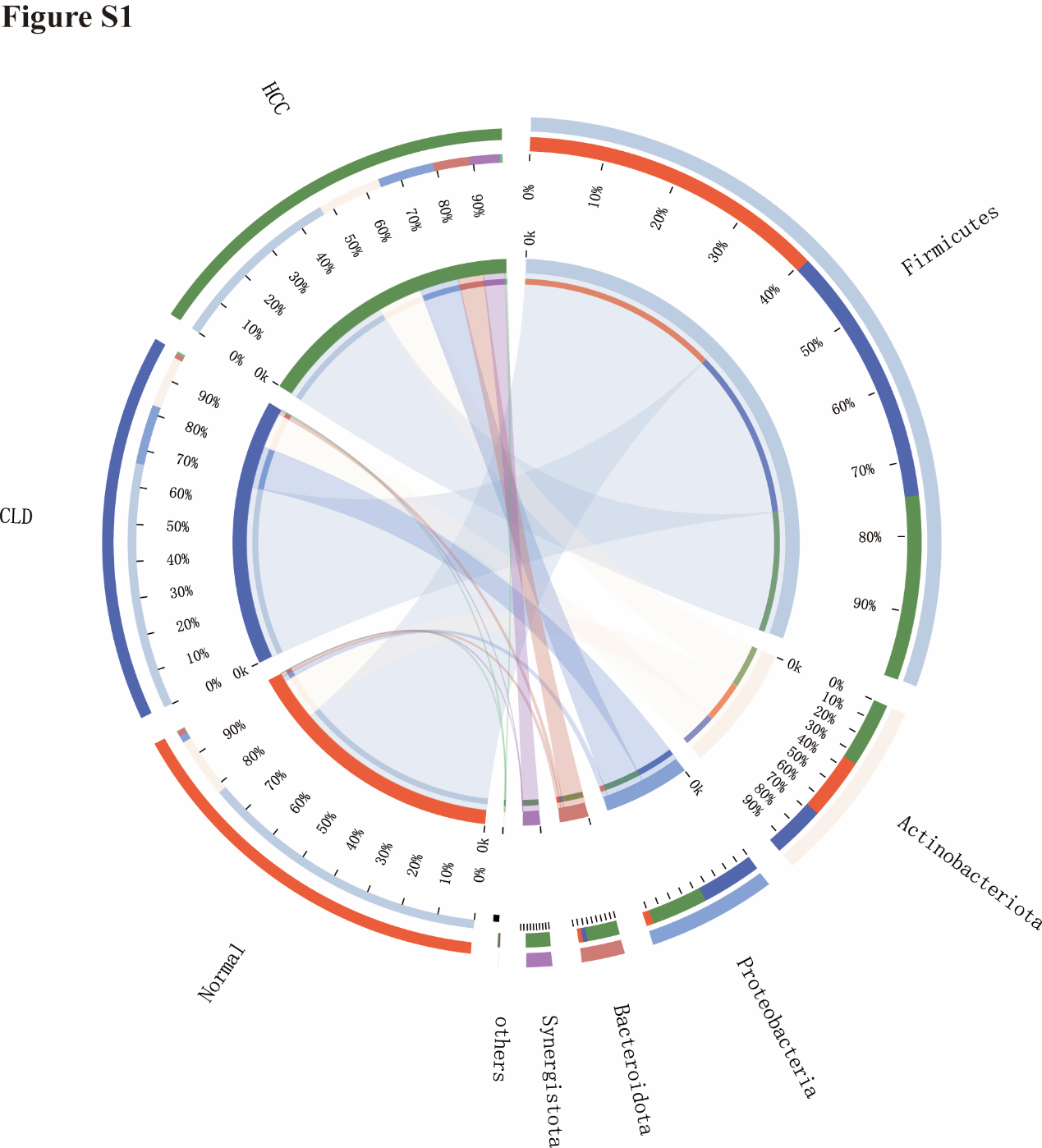
**


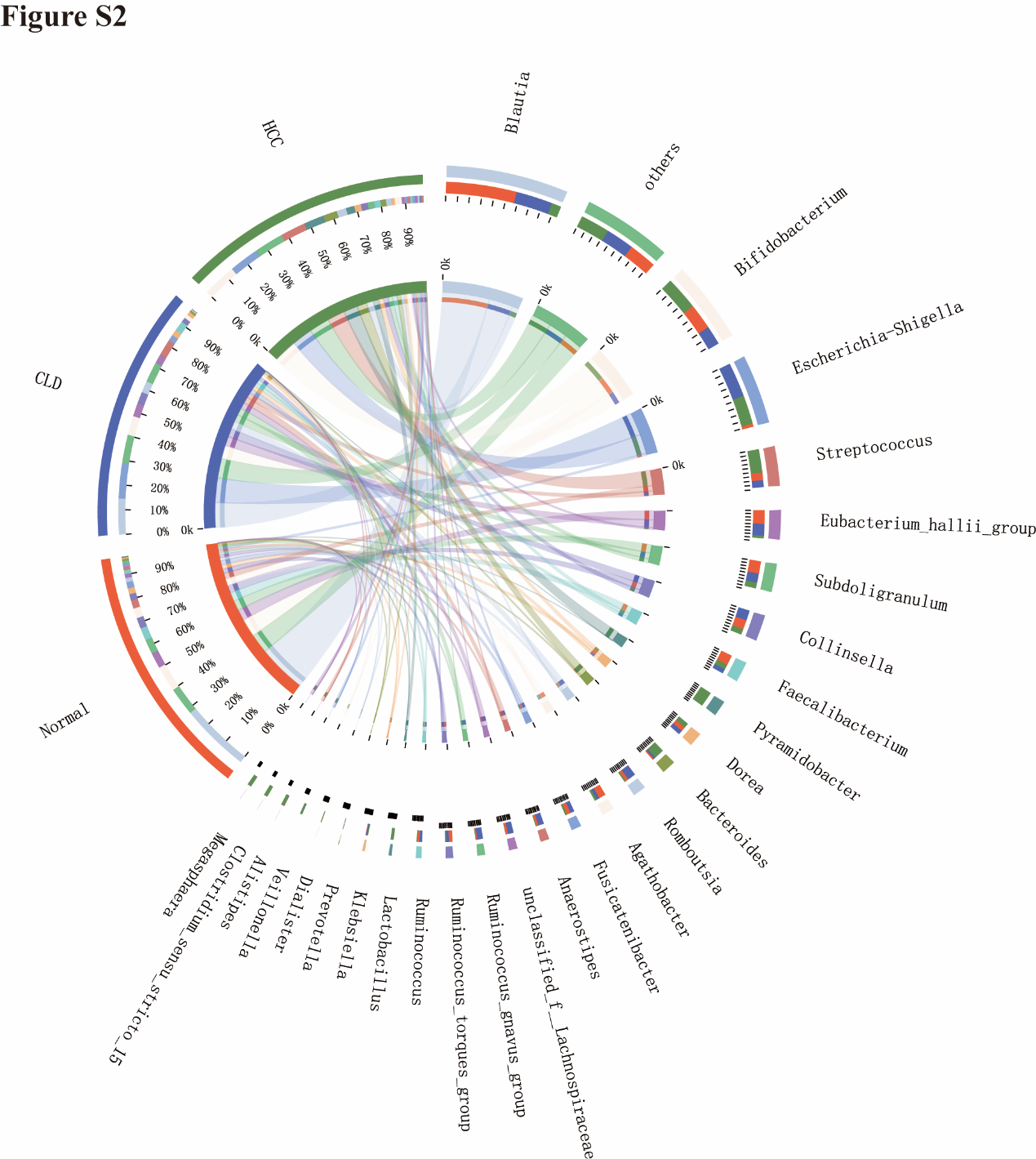

Supplement: Supplemental Material [file KBIE_A_1982273_SM9122.docx]
